# Exploring the Potential of Nitrogen Fertilizer Mixed Application to Improve Crop Yield and Nitrogen Partial Productivity: A Meta-Analysis

**DOI:** 10.3390/plants14152417

**Published:** 2025-08-04

**Authors:** Yaya Duan, Yuanbo Jiang, Yi Ling, Wenjing Chang, Minhua Yin, Yanxia Kang, Yanlin Ma, Yayu Wang, Guangping Qi, Bin Liu

**Affiliations:** 1 College of Water Conservancy and Hydrpower Engineering, Gansu Agricultural University, Lanzhou 730070, China; 1073323020376@st.gsau.edu.cn (Y.D.); 1073323010121@st.gsau.edu.cn (Y.J.); 1073324120786@st.gsau.edu.cn (Y.L.); 1073324120789@st.gsau.edu.cn (W.C.); mayl@gsau.edu.cn (Y.M.); wangyy@gsau.edu.cn (Y.W.); qigp@gsau.edu.cn (G.Q.); 1073324020394@st.gsau.edu.cn (B.L.); 2 Gansu Province Jingtai Chuan Power Irrigation Water Resource Utilization Center, Baiyin 730400, China

**Keywords:** controlled-release nitrogen fertilizer, urea, crop yield, nitrogen utilization efficiency, meta-analysis

## Abstract

Slow-release nitrogen fertilizers enhance crop production and reduce environmental pollution, but their slow nitrogen release may cause insufficient nitrogen supply in the early stages of crop growth. Mixed nitrogen fertilization (MNF), combining slow-release nitrogen fertilizer with urea, is an effective way to increase yield and income and improve nitrogen fertilizer efficiency. This study used urea alone (Urea) and slow-release nitrogen fertilizer alone (C/SRF) as controls and employed meta-analysis and a random forest model to assess MNF effects on crop yield and nitrogen partial factor productivity (PFPN), and to identify key influencing factors. Results showed that compared with urea, MNF increased crop yield by 7.42% and PFPN by 8.20%, with higher improvement rates in Northwest China, regions with an average annual temperature ≤ 20 °C, and elevations of 750–1050 m; in soils with a pH of 5.5–6.5, where 150–240 kg·ha^−1^ nitrogen with 25–35% content and an 80–100 day release period was applied, and the blending ratio was ≥0.3; and when planting rapeseed, maize, and cotton for 1–2 years. The top three influencing factors were crop type, nitrogen rate, and soil pH. Compared with C/SRF, MNF increased crop yield by 2.44% and had a non-significant increase in PFPN, with higher improvement rates in Northwest China, regions with an average annual temperature ≤ 5 °C, average annual precipitation ≤ 400 mm, and elevations of 300–900 m; in sandy soils with pH > 7.5, where 150–270 kg·ha^−1^ nitrogen with 25–30% content and a 40–80 day release period was applied, and the blending ratio was 0.4–0.7; and when planting potatoes and rapeseed for 3 years. The top three influencing factors were nitrogen rate, crop type, and average annual precipitation. In conclusion, MNF should comprehensively consider crops, regions, soil, and management. This study provides a scientific basis for optimizing slow-release nitrogen fertilizers and promoting the large-scale application of MNF in farmland.

## 1. Introduction

Nitrogen is the most critical nutrient element limiting the productivity of agricultural ecosystems [1,2]. The total annual nitrogen input into global farmland is estimated to be 200 million tons; however, utilization efficiency remains below 50%. The utilization of low nitrogen fertilizers and elevated environmental pollution are prevalent concerns in agricultural production on a global scale, especially in developing countries [3,4]. China’s annual nitrogen fertilizer application accounts for approximately one-third of the world’s total nitrogen fertilizer consumption, yet the average utilization rate of nitrogen fertilizers is only approximately 30% [5,6]. Significant nitrogen loss from farmland has been shown to lead to a number of issues concerning agricultural non-point source pollution, including water eutrophication, groundwater contamination, and greenhouse gas emissions [7,8,9,10]. In recent years, the utilization of controlled-release nitrogen fertilizers mixed with urea (MNF) has become increasingly widespread [11]. As a novel class of fertilizer, controlled-release nitrogen fertilizers (CRF) have been shown to enhance crop yield, improve efficiency, and reduce environmental impact [12,13]. The combined application of these two elements has the capacity to regulate the nitrogen cycle and maintain equilibrium within the crop-soil-atmosphere system [14,15,16]. This represents a significant measure for enhancing farmland productivity in a synergistic manner, whilst concomitantly promoting the green and sustainable development of the ecological environment [17,18].

Slow-release nitrogen fertilizer (SRF) delays nitrogen release through chemical and physical means such as low-solubility compounds, matrix encapsulation or cementation, and its mechanism depends on chemical decomposition or hydrolysis [11]. Controlled-release nitrogen fertilizer (CRF) uses polymer envelopes to form a physical barrier, and achieves precise control of nitrogen release through membrane permeation and diffusion [12]. The application of C/SRF mixed with urea (referred to as nitrogen fertilizer mixing, MNF) regulates the release rate and intensity of nitrogen, optimizing biochemical processes such as mineralization and nitrification [19]. This facilitates the conversion of more nitrogen into small-molecule inorganic nitrogen that is easily absorbed by crops, while reducing losses from nitrogen volatilization, leaching, and denitrification, thereby increasing soil available nitrogen content [17,18,19,20,21]. The improved soil nitrogen status achieved through MNF enhances the stability and diversity of soil microbial communities, optimizing soil nitrogen cycling [22]. Additionally, the sustained nutrient release characteristics of MNF effectively regulate soil nitrogen distribution and movement, reducing nitrogen accumulation in deeper soil layers and lowering risks of soil acidification, erosion, and groundwater pollution [23,24]. While improving the soil nutrient environment, MNF promotes crop nutrient absorption and growth [25]. The nitrogen release patterns of MNF align closely with crop growth requirements, significantly improving root morphology, distribution, and vitality, enhancing crop nutrient absorption and conversion rates, and facilitating the continuous transport and accumulation of nutrients to the crop canopy [26,27,28]. Additionally, MNF increases leaf chlorophyll content, photosynthetic rates, and antioxidant enzyme activity, delays plant senescence and leaf lignification, and enhances crop biomass accumulation and stress tolerance [29,30]. Furthermore, MNF regulates organic matter distribution, promotes crop flower bud differentiation, flowering, and seed set, increases effective panicle number, grain weight, and seed set rate, and enhances nutrient content such as vitamins, proteins, and amino acids, thereby improving crop yield and quality [31,32,33]. MNF also improves crop utilization efficiency of deep soil nutrients and resources such as light, heat, and air by optimizing root system structure and leaf function [31,33]. However, the extant research has not yet established universal standards for nitrogen fertilizer blending (C/SRF: Urea). Research has identified the optimal blending ratio for corn as 2.5:7.5, while rice exhibits optimal performance at a 4:6 ratio [34,35]. Winter wheat achieves peak yield and economic benefits at approximately 7:3, and potatoes demonstrate significant environmental benefits at a 1:1 ratio [36,37]. Moreover, research findings have indicated that the release of nitrogen from nitrogen fertilizer demonstrates a high degree of environmental sensitivity [38]. In cold regions, the release of nitrogen is almost nonexistent, while in flooded environments, the risk of nitrogen loss is increased. In arid regions, the rupture of nitrogen fertilizer coatings can be hindered by drought conditions, thereby reducing nitrogen release rates [39,40]. Concurrently, the regional differentiation characteristics of the nitrogen fertilizer mixing effects are also of considerable significance. Research conducted in the cold-temperate regions of the United States and Japan has indicated that the utilization of controlled-release nitrogen fertilizer has resulted in yield reductions ranging from 10.03% to 17.70% for corn and winter wheat [41]. Conversely, in the context of tropical rice cultivation, the application of nitrogen fertilizer has been observed to enhance yields by 7.43–22.84% [42]. It is evident that the effects of nitrogen fertilizer mixing exhibit high complexity and context dependency. Quantitative analysis of the interactive mechanisms of the “crop-environment-management” system and identification of context-appropriate nitrogen fertilizer mixing methods remain critical scientific challenges for achieving stable and efficient agricultural production and environmental sustainability goals.

In summary, the field effects of MNF vary significantly across studies, with some results even being contradictory. However, the primary sources of these differences remain unclear. Does the effect of MNF vary with crop type, region, and soil conditions? Do fertilizer type, application rate, and mixing ratio influence the effects of MNF? How can the advantages of MNF be maximized? These questions are difficult to answer through individual studies. In view of this, this study selected key parameters that directly reflect the production effects of MNF on crops—yield and nitrogen partial productivity (PFPN). By collecting publicly available field trial data on MNF in China from both domestic and international sources, and conducting a meta-analysis and random forest model using urea application and CRF application alone as controls, the objectives were to: (1) quantify the overall effects of MNF on crop yield and PFPN; (2) identify the main influencing factors and their importance for MNF on crop yield and PFPN; and (3) explore the yield-increasing and efficiency-enhancing potential of MNF. This research combines meta-analysis and random forest models to quantify the yield and PFPN enhancement effects of MNF, enabling high-yield, high-quality crop cultivation methods and providing a basis for further improving crop production levels and promoting green, high-quality agricultural development.

## 2. Results

### 2.1. Statistical Analysis of the Effects of MNF on Crop Yield and PFPN

#### 2.1.1. Data Distribution

**Figure 1:**
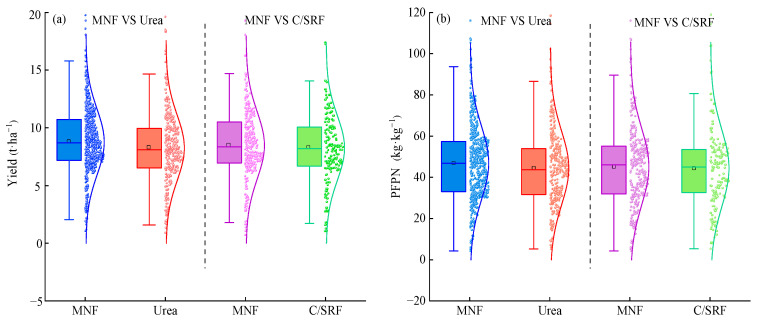
Crop yield and PFPN under MNF, urea and C/SRF. (**a**) MNF vs. Urea, (**b**) MNF vs. C/SRF. The upper edge, internal horizontal line, lower edge and black box indicate the 3/4 quartile (Q_3_), median, 1/4 quartile (Q_1_), and mean, respectively. The lower and upper ends of the vertical lines indicate the minimum and maximum values within the normal range, respectively. The curves in the plots are the normal distribution lines, and the dots in the plots represent the sample values.

Due to variations in climatic conditions and management practices, both crop yield and PFPN exhibited significant variability across the two combinations (Figure 1). For the combination of MNF and urea, the crop yield and PFPN for the former ranged from 0.1 to 111.8 t·ha^−1^ and 0.5 to 614.9 kg·kg^−1^, respectively. For the latter, the crop yield and PFPN ranged from 0.1 to 105.4 t·ha^−1^ and 0.5 to 602.3 kg·kg^−1^, respectively. These results indicate that MNF can improve both crop yield and PFPN compared to Urea. For the combination of MNF and C/SRF, the crop yield and PFPN for the former ranged from 0.6 to 76.6 t·ha^−1^ and 4.3 to 230.6 kg·kg^−1^, respectively. For the latter, the crop yield and PFPN ranged from 0.9 to 77.8 t·ha^−1^ and 5.3 to 200.8 kg·kg^−1^, respectively. It can be seen that some crop yield and PFPN of MNF application are lower than those of C/SRF application.

#### 2.1.2. Overall Effect

**Figure 2:**
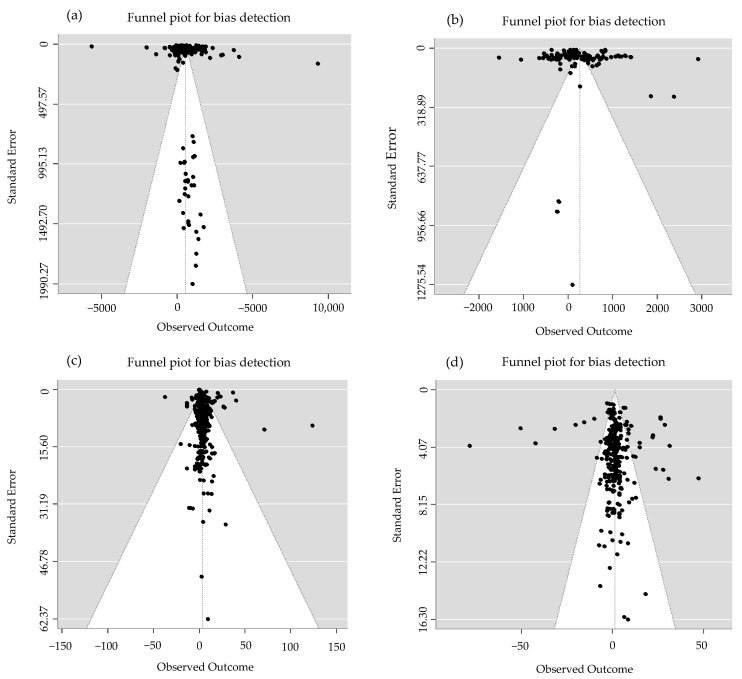
Inverted funnel by publication bias test. (**a**,**c**) represent the yield and PFPN of MN vs. urea, respectively, while (**b**,**d**) represent the yield and PFPN of MN vs. C/SRF, respectively. The points in the figure represent the included studies, the vertical line represents the ideal effect line, and the two diagonal lines represent the 95% confidence interval.

The overall effect sizes of MNF on crop yield and PFPN were calculated (Table 1). The heterogeneity test results were significant (*p* < 0.0001), indicating heterogeneity among the studies; therefore, a random effect model was used. Overall, compared to Urea, MNF significantly increased crop yield by 7.42% (95% CI: 6.49~8.36%) and PFPN by 8.20% (95% CI: 7.22~9.87%). Compared to C/SRF, MNF significantly increased crop yield by 2.44% (95% CI: 1.24–3.66%), while the increase in PFPN was not significant (0.24%, 95% CI: −0.63~3.20%). After publication bias testing, the P_B_ values for yield and PFPN for MN vs. urea and MN vs. C/SRF were both greater than 0.05 (Table 1), and the sample data points were evenly distributed on both sides of the funnel (Figure 2), indicating that there was no publication bias.

### 2.2. Analysis of Factors Affecting the Effects of MNF on Crop Yield

#### 2.2.1. Characteristics of Nitrogen Fertilizer

**Figure 3:**
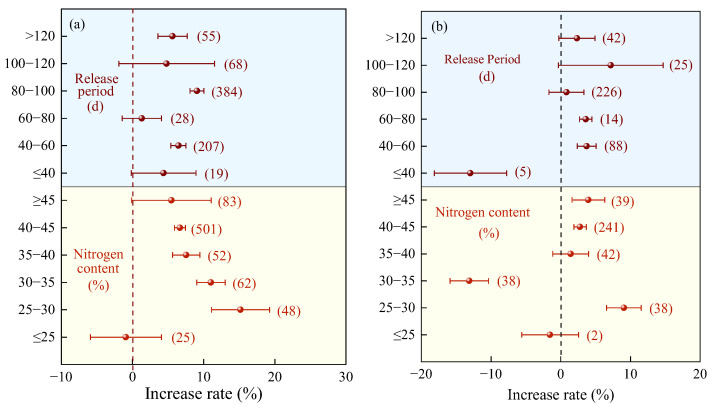
Effects of the characteristics of nitrogen fertilizer on the yield increase rate. (**a**) MNF vs. urea, (**b**) MNF vs. C/SRF. Error bars represent the 95% confidence interval, and the numbers in the figures indicate the corresponding sample size for each group.

Compared with the urea, the yield increase in crops treated with mixed nitrogen fertilizers shows a trend of first increasing and then decreasing, influenced by nitrogen content, mixing ratio, and nitrogen application rate, with an optimal range existing (Figure 3). The highest yield increase was observed at a nitrogen content of 25–30% (average 15.17%, Figure 3a), but there was no significant difference compared to 30–35%. When the nitrogen content was ≤25% or ≥45%, the yield increase was not significant. There was no obvious pattern in the yield increase rate related to the nitrogen release period, but the effect was better when the release period was 80–100 days (average 9.06%, Figure 3a). The yield increase rate of mixed CRF (average 9.20%, Figure 3a) was higher than that of SRF (average 6.77%), but the difference between the two was not significant.

Similarly, compared to the application of C/SRF alone, the highest crop yield increase (average 9.08%) was observed when MNF and the nitrogen content were between 25% and 30% (Figure 3b), followed by nitrogen content ≥40%. When nitrogen content was ≤25% or 35–40%, the effect was not significant, and when nitrogen content was 30–35%, crop yield was significantly reduced. When the nitrogen release period was ≤40 days, MNF significantly reduced yields by 12.96% (Figure 3b); between 40 and 80 days, average yield increases were 3.60–3.73%; and when the release period was ≥80 days, the effect was not significant.

#### 2.2.2. Nitrogen Fertilizer Management

**Figure 4:**
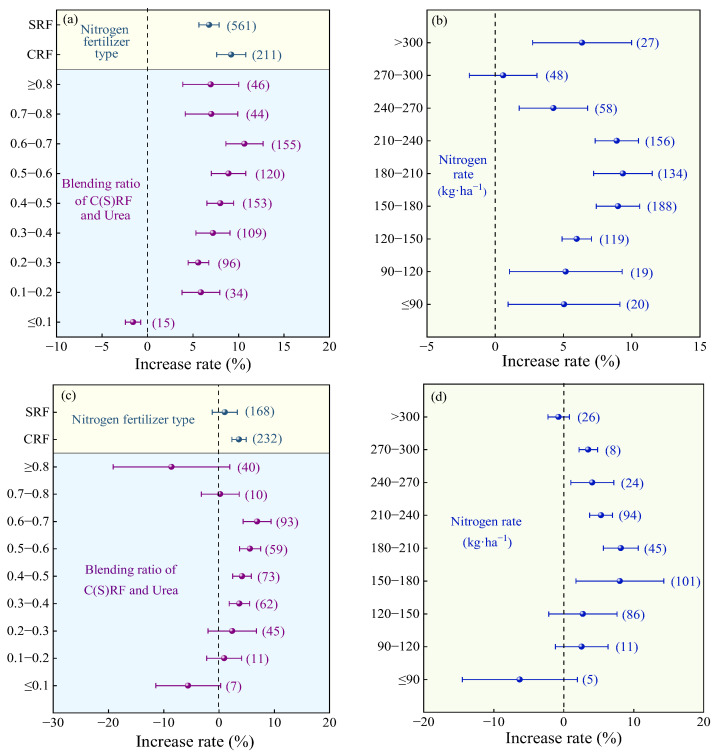
Effects of the nitrogen fertilizer management on the crop yield increase rate. (**a**,**b**) MNF vs. urea, and (**c**,**d**) MNF vs. C/SRF. Error lines and dots indicate 95% confidence intervals and average improvement rates, respectively, and the numbers to the right of the error lines indicate the number of samples in the corresponding subgroups.

Compared with the urea, the highest yield increase rate was observed at a mixing ratio of 0.6–0.7 (average 10.66%, Figure 4a), with no significant difference compared to 0.3–0.6 and >0.7. When the mixing ratio was ≤0.1, the yield significantly decreased. The highest yield increase rate was observed when the nitrogen application rate was 180–210 kg·ha^−1^ (average 9.35%, Figure 4b), with no significant difference compared to 150–180 kg·ha^−1^ and 210–240 kg·ha^−1^. Compared with C/SRF alone, when the mixing ratio was 0.6–0.7, average yield increased by 6.90% (Figure 4c), but there was no significant difference compared to 0.3–0.6. When the mixing ratio was ≤0.3 or >0.7, the yield increase effect was not significant. The application of CRF fertilizer significantly increased crop yield (average +3.62%), while the application of SRF had no significant effect (Figure 4c). The yield increase from nitrogen fertilizer application was highest at nitrogen application rates of 180–210 kg·ha^−1^ (average 8.17%, Figure 4d), but there was no significant difference compared to 150–180 kg·ha^−1^ and 210–270 kg·ha^−1^. When the nitrogen application rate was <150 kg·ha^−1^ or >300 kg·ha^−1^, the yield-increasing effect of nitrogen fertilizer application was not significant.

#### 2.2.3. Regional Factor

**Figure 5:**
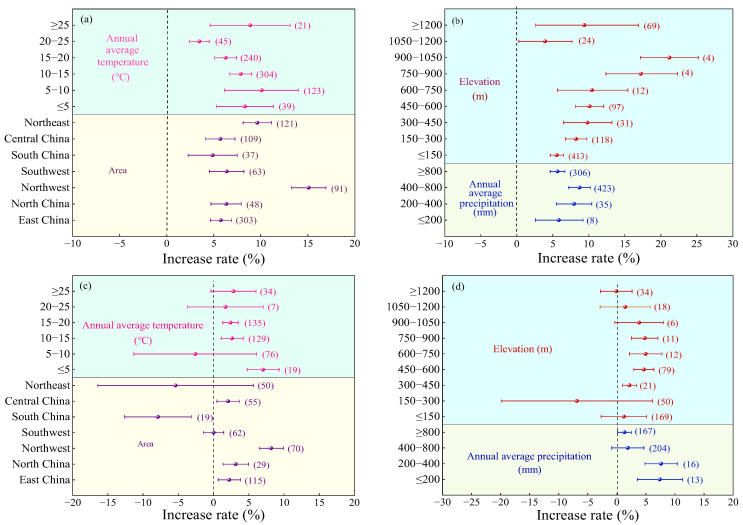
Effects of the regional factors on the yield increase rate. (**a**,**b**) MNF vs. urea, and (**c**,**d**) MNF vs. C/SRF. Error lines and dots indicate 95% confidence intervals and average improvement rates, respectively, and the numbers to the right of the error lines indicate the number of samples in the corresponding subgroups.

Compared to urea (Figure 5a), the crop yield increase in MNF was significantly higher in Northwest China (mean 15.10%, 95% CI: 13.28~16.92%), followed by Northeast China (mean 9.62%, 95% CI: 8.12~11.12%), East China (5.76%), North China (6.33%), Southwest China (6.37%), South China (4.88%), and Central China (5.7%), with an average yield increase that was lower and not significantly different. With the increase in AAT (Figure 5a), AAP (Figure 5b), and altitude (Figure 5b), the yield rate of crops MNF first increases and then decreases. When the AAT was 5–10 °C (average 10.09%, 95% CI: 6.17~14.01%), the AAP was 400–800 mm (average 8.69%, 95% CI: 7.22~10.16%), and the altitude was 900–1050 m (average 21.19%, 95% CI: 17.19~25.19%), the yield increase rate was the highest.

Compared to C/SRF, the yield increase rate of MNF was significantly higher in Northwest China (8.18%, 95% CI: 6.47~9.89%, Figure 5c) than in other regions, followed by East China (2.23%), North China (3.14%), and Central China (2.07%). The yield increase effects in Southwest China and Northeast China were not significant. In South China, MNF significantly reduced crop yield by 7.88% (95% CI: −12.61~3.15%). When the AAT was <10 °C or ≥20 °C, the yield effect of MNF was not significant. When the AAT was ≥10 °C and <20 °C, the average yield increase rate ranged from 2.40% to 2.63% (Figure 5c). When the AAP was ≥400 mm, the yield effect of MNF was not significant. When the AAP was <400 mm, the average yield increase rate ranged from 7.62% to 7.40% (Figure 5d). The effect of MNF on PFPN showed a trend of first increasing and then decreasing with increasing elevation. The highest average increase rate was observed at an elevation of 600–750 m (4.96%, 95% CI: 2.16~7.76%), but the difference was not significant compared to elevations of 300–600 m and 750–900 m (Figure 5d).

#### 2.2.4. Soil Factor

**Figure 6:**
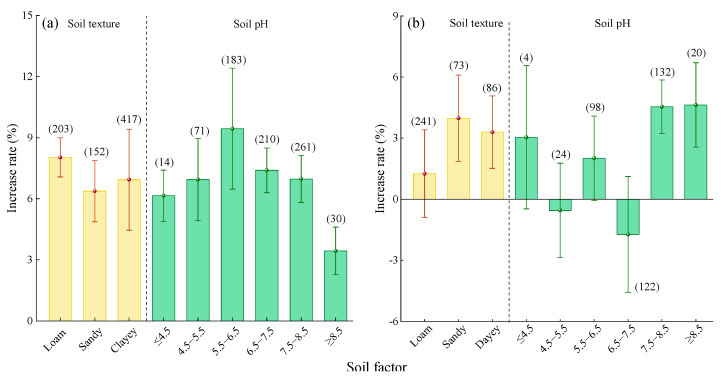
Effects of the soil factors on the yield increase rate. (**a**) MNF vs. Urea, (**b**) MNF vs. C/SRF. Error bars represent the 95% confidence interval, and the numbers in the figures indicate the corresponding sample size for each group.

Compared to Urea, there was no significant difference in the yield increase effects of MNF across different soil textures (Figure 6a). Among the soil types, the highest average yield increase rate was observed for loam (8.03%, 95% CI: 7.07~8.99%), followed by clay (6.94%, 95% CI: 4.92~8.96%) and sand (6.37%, 95% CI: 4.87~7.87%). The yield increase rate of MNF showed a trend of first increasing and then decreasing with increasing soil pH (Figure 6a). The highest yield increase rate was observed when the soil pH was 5.5–6.5 (9.44%, 95% CI: 6.47~12.41%), but the difference was not significant compared to soils with pH ≤ 5.5 or 6.5 ≤ pH < 8.5. When the soil pH was ≥8.5, the yield increase rate significantly decreased (3.44%, 95% CI: 2.27~4.61%).

Compared to C/SRF (Figure 6b), the average yield increase rates of MNF were 3.98% (95% CI: 1.86~6.1%) for sandy soil, 3.30% (95% CI: 1.52–5.08%) for clay, and 1.25% (95% CI: −0.9~3.4%) for loam (Figure 6b). When the soil pH was <7.5, there was no clear pattern in the yield effect of MNF. When the soil pH was ≥7.5, the yield increase rate stabilized between 4.54% and 4.63%.

#### 2.2.5. Crop Type

**Figure 7:**
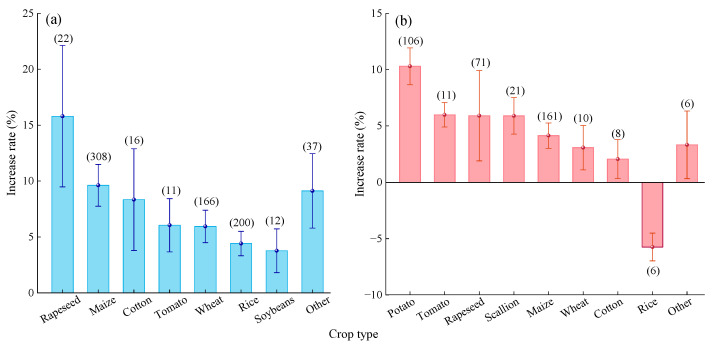
Effects of the crop factor on the yield increase rate. (**a**) MNF vs. urea, (**b**) MNF vs. C/SRF. Error bars represent the 95% confidence interval, and the numbers in the figures indicate the corresponding sample size for each group.

Compared to urea (Figure 7a), MNF had the highest yield increase effect on rapeseed (15.80%, 95% CI: 9.48~22.12%). However, the differences were not significant compared to maize (9.62%, 95% CI: 7.75~11.49%), cotton (8.34%, 95% CI: 3.79~12.89%), and other crops (9.12%, 95% CI: 5.78~12.45%). The yield increase effects for tomato (6.05%) and wheat (5.94%) were moderate, while those for rice (4.41%) and soybean (3.77%) were relatively low.

Compared to C/SRF (Figure 7b), MNF resulted in the highest yield increase effect for potato (10.3%, 95% CI: 8.67–11.93%), although the difference was not significant compared to rapeseed (5.91%, 95% CI: 1.9–9.92%). The yield increase effects for green onion (5.90%) and tomato (5.99%) were moderate. In contrast, the yield increase effects for wheat (3.08%), cotton (2.06%), maize (4.15%), and other crops (3.33%) were relatively low. Notably, MNF resulted in a significant 5.74% decrease in the average yield of rice.

#### 2.2.6. Time Factor

**Figure 8:**
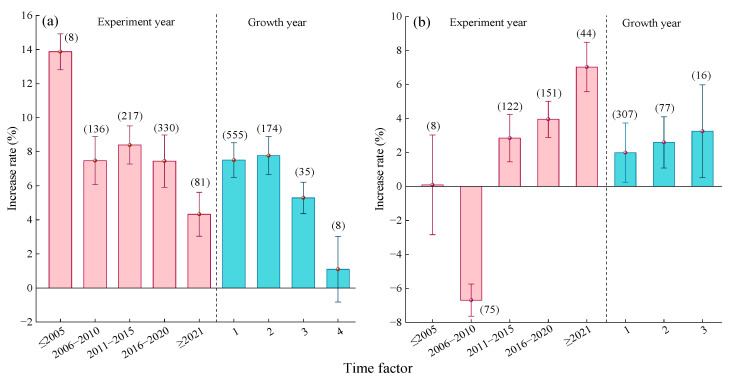
Effects of the time factor on the yield increase rate. (**a**) MNF vs. urea, (**b**) MNF vs. C/SRF. Error bars represent the 95% confidence interval, and the numbers in the figures indicate the corresponding sample size for each group.

Compared to urea (Figure 8a), before 2005, the average yield increase rate from MNF was 13.87% (95% CI: 12.12~15.62%), which was significantly higher than in other years. After 2006, the yield increase rate showed a trend of first increasing and then decreasing, with the highest average yield increase rate observed during 2011–2015 (4.33%, 95% CI: 3.04~5.62%, Figure 8a). With increasing planting duration, the average yield increase effect of MNF also exhibited a trend of first increasing and then decreasing. Specifically, the yield increase rates for crops with planting durations of 1 year (7.51%, 95% CI: 6.49~8.53%) and 2 years (7.77%, 95% CI: 6.65~8.89%) showed no significant difference. When the planting duration reached 4 years, the yield increase effect of MNF was not significant.

Compared to C/SRF (Figure 8b), before 2005, the yield effect of MNF was not significant (Figure 8b). Between 2006 and 2010, the average yield significantly decreased by 6.69%. After 2011, the average yield increase rate showed an upward trend, reaching 7.03% (95% CI: 5.58~8.48%) after 2021. With increasing planting duration, the yield increase effect of MNF exhibited an upward trend. When the planting duration was 3 years, the average yield increase rate was 3.25% (95% CI: 0.52~5.98%), but the difference was not significant compared to planting durations of 1 year and 2 years.

### 2.3. Analysis of Factors Affecting the Effects of MNF on Crop PFPN

#### 2.3.1. Characteristics of Nitrogen Fertilizer

**Figure 9:**
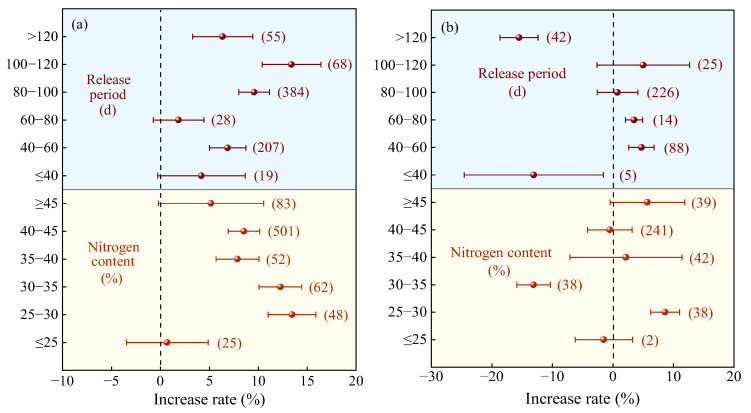
Effects of the characteristics of nitrogen fertilizer on PFPN increase rate. (**a**) MNF vs. urea, (**b**) MNF vs. C/SRF. Error bars represent the 95% confidence interval, and the numbers in the figures indicate the corresponding sample size for each group.

Compared with urea (Figure 9a), when the nitrogen content was 25–30%, the average PFPN increase rate of MNF was the highest (13.45%, 95% CI: 11.02~15.88%), but the difference was not significant compared to 30–35% nitrogen content (12.26%, 95% CI: 10.07~14.45%). The PFPN increase effect was not significant when the nitrogen content was ≤25% or ≥45%. The average PFPN increase rate of MNF showed an overall trend of first increasing and then decreasing with increasing nitrogen release period (Figure 9a). The highest average increase rate was observed for a nitrogen release period of 100–120 days (13.40%, 95% CI: 10.39~16.41%), but the difference was not significant compared to 80–100 days (9.58%, 95% CI: 8.01–11.15%).

Compared with C/SRF (Figure 9b), when the nitrogen content was 25–30%, the average PFPN increase rate of MNF was significantly 8.62% (95% CI: 6.24~11.00%). When the nitrogen content was ≤25% or ≥30%, the effect of MNF on PFPN was either not significant or showed a significant decrease. The PFPN increased effect of MNF showed a trend of first increasing and then decreasing with increasing nitrogen release period (Figure 9b). When the nitrogen release period was 40–80 days, the average PFPN increase rate was significantly 3.94–4.72%. When the nitrogen release period was ≤40 days or >80 days, the effect on PFPN was either not significant or showed a significant decrease.

#### 2.3.2. Nitrogen Fertilizer Management

**Figure 10:**
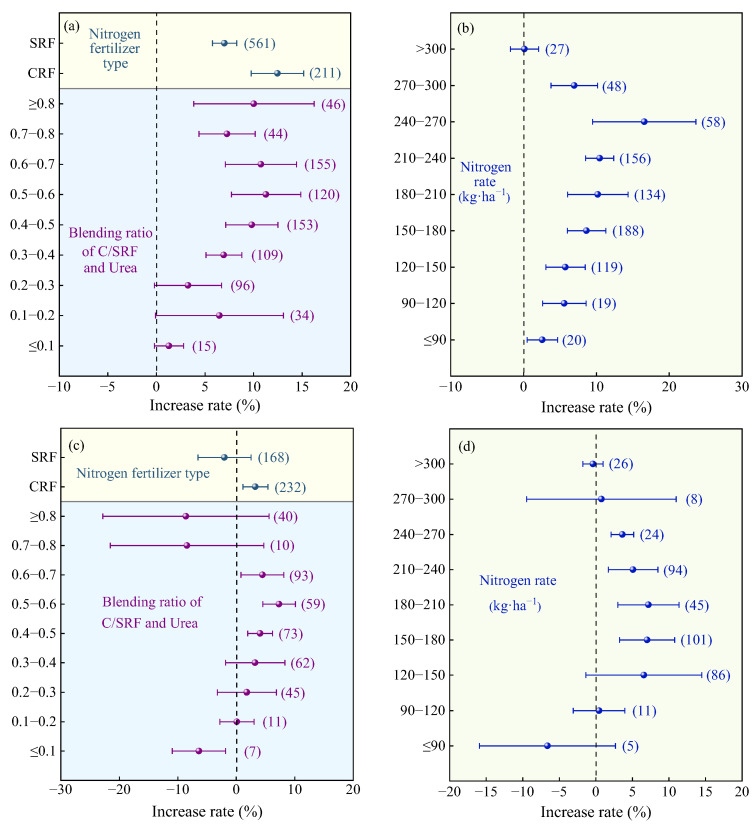
Effects of the management factor on PFPN. (**a**,**b**) MNF vs. urea, and (**c**,**d**) MNF vs. C/SRF. Error lines and dots indicate 95% confidence intervals and average improvement rates, respectively, and the numbers to the right of the error lines indicate the number of samples in the corresponding subgroups.

Compared with urea, with an increasing blending ratio, the PFPN increase rate exhibited a trend of first increasing and then decreasing (Figure 10a). The highest average increase rate was observed when the blending ratio was 0.5–0.6 (11.29%, 95% CI: 7.72~14.86%), with no significant difference compared to 0.3–0.5 and > 0.6. The average PFPN increase rate for MNF with CRF (12.47%, 95% CI: 9.77~15.77%) was significantly higher than that for SRF (7.01%, 95% CI: 5.77~8.25%, Figure 10a). The PFPN increase rate of MNF showed a trend of first increasing and then decreasing with increasing N rate (Figure 10b). The highest average increase rate was observed at an N rate of 240–270 kg·ha^−1^ (16.58%, 95% CI: 8.57~24.59%), but the difference was not significant compared to 150–240 kg·ha^−1^ and 270–300 kg·ha^−1^. When the N rate was >300 kg·ha^−1^, the PFPN increase effect was not significant.

Compared with C/SRF, MNF with CRF significantly increased PFPN by 3.27% (95% CI: 1.14~5.40%, Figure 10c), while blending with SRF had no significant effect on PFPN. The average PFPN increase rate exhibited a trend of first increasing and then decreasing with increasing blending ratio (Figure 10c) and N rate (Figure 10d). When the blending ratio was 0.5–0.6, the highest average PFPN increase rate was 7.31% (95% CI: 4.51~10.11%), but the difference was not significant compared to blending ratios of 0.4–0.5 (4.09%) and 0.6–0.7 (4.47%). Similarly, when the N rate was 180–210 kg·ha^−1^, the highest average PFPN increase rate was (7.20%, 95% CI: 3.02~11.38%), but the difference was not significant compared to application rates of 150–180 kg·ha^−1^ and 210–270 kg·ha^−1^.

#### 2.3.3. Regional Factor

**Figure 11:**
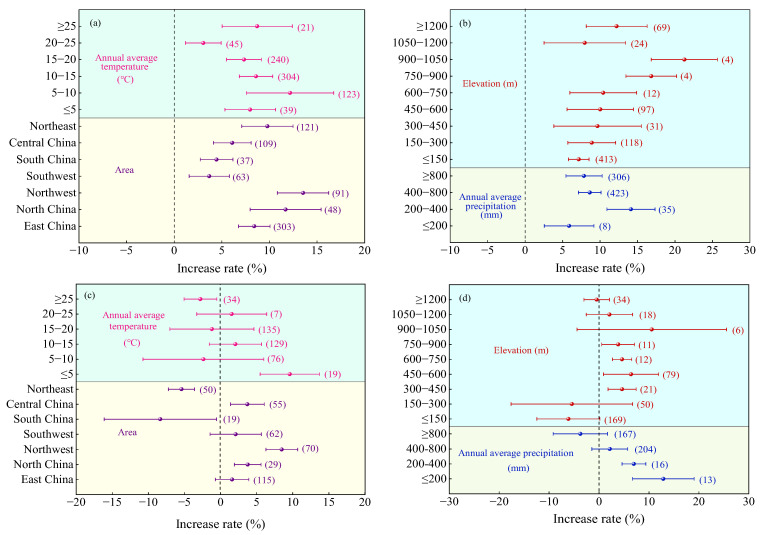
Effects of the regional factors on PFPN. (**a**,**b**) MNF vs. urea, and (**c**,**d**) MNF vs. C/SRF. Error lines and dots indicate 95% confidence intervals and average improvement rates, respectively, and the numbers to the right of the error lines indicate the number of samples in the corresponding subgroups.

Compared with urea (Figure 11a), the highest average PFPN increase rate of MNF was observed in the Northwest (13.53%, 95% CI: 10.83~16.23%), but the difference was not significant compared to North China (11.7%, 95% CI: 7.97~15.43%) and the Northeast (9.79%, 95% CI: 7.10~12.48%). The average increase rates in East China (8.41%) and Central China (6.10%) were moderate, while South China (4.45%) and the Southwest (3.68%) had the lowest increase rates. When the AAT was 5~10 °C, the highest PFPN increase rate was observed (12.17%, 95% CI: 7.61~16.73%, Figure 11a), with no significant differences compared to AAT of ≤5 °C, 10–20 °C, and ≥25 °C. The lowest PFPN increase rate was observed when the AAT was 20–25 °C (3.06%, 95% CI: 1.19~4.93%). With increasing AAP and elevation, the PFPN increase rate of MNF showed a trend of first increasing and then decreasing (Figure 11b). The highest PFPN increase rate was observed when the AAP was 200–400 mm (14.13%, 95% CI: 10.92~17.34%), which was significantly higher than when the AAP was ≤200 mm or >400 mm. For elevation (Figure 11b), the highest PFPN increase rate was observed at 900–1050 m (21.26%, 95% CI: 16.84~25.68%), with no significant difference compared to 750–900 m (16.84%, 95% CI: 13.45~20.23%).

Compared with C/SRF (Figure 11c), the highest PFPN increase rate of MNF was observed in Northwest (8.48%, 95% CI: 6.28~10.68%), followed by North China (3.76%) and Central China (3.71%). No significant effects were observed in East China and the Southwest, while significant decreases were recorded in South China and the Northeast. The PFPN increase rate decreased with increasing AAT and AAP (Figure 11c,d). When the AAT was ≤5 °C and the AAP was ≤400 mm, MNF significantly increased PFPN by 9.59% and 6.99–12.87%, respectively. When the AAT was 5–25 °C and the AAP was >400 mm, the effect of MNF on PFPN was not significant. The PFPN increase rate exhibited a trend of first increasing and then decreasing with rising elevation (Figure 11d). The highest average increase rate was observed at an elevation of 450–600 m (6.42%, 95% CI: 0.89~11.95%), but the difference was not significant compared to elevations of 300–450 m and 600–900 m.

#### 2.3.4. Soil Factor

**Figure 12:**
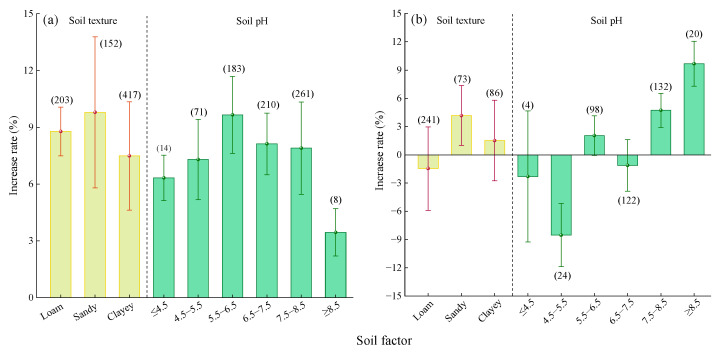
Effects of the soil factor on the crop PFPN increase rate. (**a**) MNF vs. urea, (**b**) MNF vs. C/SRF. Error bars represent the 95% confidence interval, and the numbers in the figures indicate the corresponding sample size for each group.

Compared with urea (Figure 12a), the average PFPN increase rates of MNF were 9.79% (95% CI: 5.8~13.78%) in sandy soil, 8.78% (95% CI: 7.49~10.07%) in loam, and 7.49% (95% CI: 4.63~10.35%) in clay, with no significant differences among the three soil types. The PFPN increase rate showed a trend of first increasing and then decreasing with rising soil pH (Figure 12a). The highest average increase rate was observed when 5.5 ≤ pH < 6.5 (9.66%, 95% CI: 7.63~11.69%). When pH ≥ 8.5, the PFPN increase rate was significantly the lowest (3.45%, 95% CI: 2.2~4.7%).

Compared with C/SRF (Figure 12b), MNF had no significant effect on PFPN in loam and clay, but in sandy soil, it significantly increased PFPN by 4.17% (95% CI: 0.99~7.35%). When the soil pH was ≤7.5, the effect of MNF on PFPN was either not significant or showed a significant decrease (Figure 12b). When the soil pH was >7.5, the PFPN increase rate showed a significant upward trend. The highest average increase rate was observed when the soil pH was 7.5–8.5 (4.73%, 95% CI: 2.91~6.55%), while at pH ≥ 8.5, the average increase rate was 9.67% (95% CI: 7.69~11.65%).

#### 2.3.5. Crop Type

**Figure 13:**
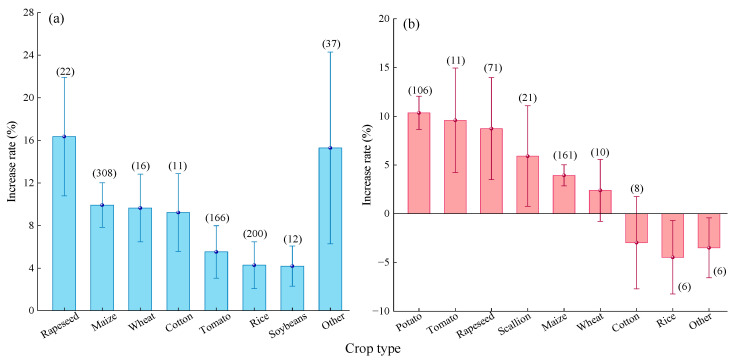
Effects of the crop factor on the crop PFPN increase rate. (**a**) MNF vs. urea, (**b**) MNF vs. C/SRF. Error bars represent the 95% confidence interval, and the numbers in the figures indicate the corresponding sample size for each group.

Compared with Urea, MNF improved PFPN to varying degrees across the eight crop types studied (Figure 13a). The highest average improvement was observed for rapeseed (16.36%, 95% CI: 7.36~25.36%), although the difference was not significant compared to maize (9.92%, 95% CI: 7.83~12.01%), wheat (9.64%, 95% CI: 6.46~12.82%), cotton (9.22%, 95% CI: 5.56~12.88%), and other crops (15.29%, 95% CI: 9.72~20.86%). The average improvement rates for tomato (5.52%), rice (4.27%), and soybean (4.19%) were relatively low, with no significant differences among them.

Compared with C/SRF (Figure 13b), MNF resulted in the highest average PFPN increase rate for potato (10.34%, 95% CI: 8.63~12.05%), but the difference was not significant compared to tomato (9.58%, 95% CI: 4.22~14.94%) and rapeseed (8.73%, 95% CI: 3.49~13.97%). The average PFPN increase rate for maize was only 3.94% (95% CI: 2.87~5.01%). The effects of MNF on the PFPN of green onion, wheat, and cotton were not significant. However, MNF significantly reduced the PFPN of rice and other crops by 3.50–4.47%.

#### 2.3.6. Time Factor

**Figure 14:**
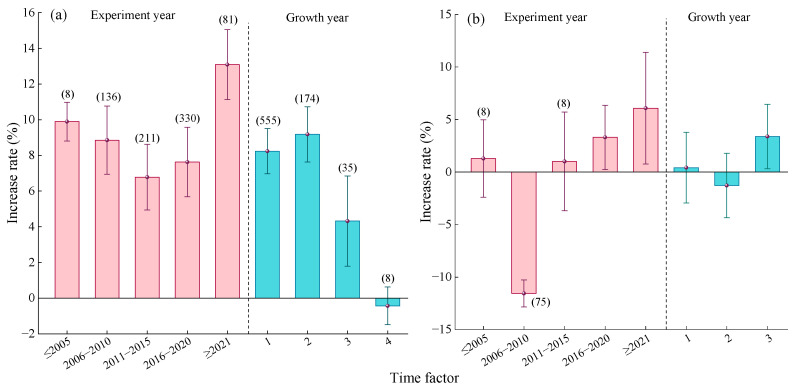
Effects of the time factor on the crop PFPN increase rate. (**a**) MNF vs. urea, (**b**) MNF vs. C/SRF. Error bars represent the 95% confidence interval, and the numbers in the figures indicate the corresponding sample size for each group.

Compared with urea, with advancing experimental years, the PFPN increase rate of MNF showed a trend of first decreasing and then increasing (Figure 14a). The lowest increase rate was observed during 2011–2015 (6.78%, 95% CI: 4.94~8.62%), while the highest increase rate was recorded after 2021 (13.09%, 95% CI: 11.13~15.05%). The PFPN increase rate was relatively high for crops with a planting duration of 1 year (8.24%, 95% CI: 6.97~9.51%) and 2 years (9.18%, 95% CI: 7.63~10.73%). When the planting duration was 3 years, the PFPN increase rate significantly decreased to 4.32% (95% CI: 1.79~6.85%). For a planting duration of 4 years, the effect of MNF on PFPN was not significant (Figure 14a).

Compared with C/SRF (Figure 14b), before 2015, the effect of MNF on crop PFPN was either not significant or showed a significant decrease. After 2016, the PFPN increase rate exhibited a stable upward trend. The average increase rate during 2016–2020 was 3.31% (95% CI: 0.25~6.37%), while after 2021, it was 6.08% (95% CI: 0.76~11.4%). MNF had no significant effect on the PFPN of crops with a planting duration of 1 year or 2 years (Figure 14b), but when the planting duration was 3 years, the average PFPN increase rate was significantly 3.39% (95% CI: 0.31~6.47%).

### 2.4. Analysis of the Importance of Factors Affecting Crop Yield and PFPN Under MNF

**Figure 15:**
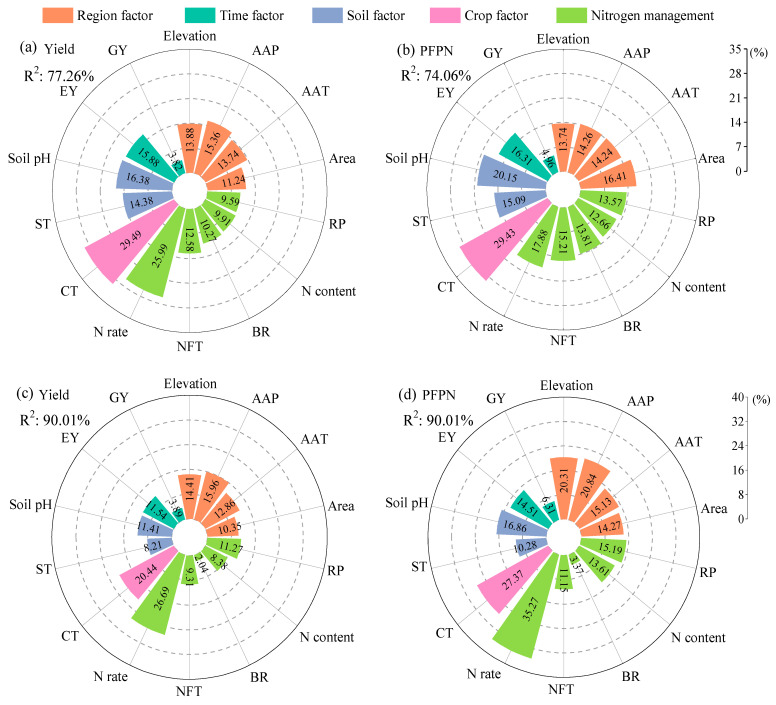
Average importance of the factors in affecting changes in crop yield and PFPN. (**a**,**b**) represent the yield and PFPN of MN vs. urea, respectively, while (**c**,**d**) represent the yield and PFPN of MN vs. C/SRF, respectively. EY: Experiment year; GY: Growth year; AAP: Annual average precipitation; AAT: Annual average temperature; ST: Soil texture; NFT: Nitrogen fertilizer texture; RP: Release period; BR: Blending ratio of MN; CT: Crop type.

A random forest model was used to conduct an importance analysis of 14 factors influencing crop yield and nitrogen fertilizer productivity effects under mixed nitrogen fertilizer application. The R^2^ values ranged from 74.06% to 90.01%, indicating a high explanatory power of the model. The results indicate that, compared to urea application alone, the top three factors influencing crop yield are crop type (29.49%, Figure 15a), nitrogen application rate (25.99%), and soil pH (16.38%). The top three factors influencing crop nitrogen fertilizer productivity were crop type (29.43%, Figure 15b), soil pH (20.15%), and nitrogen application rate (17.88%). Compared with the application of controlled-release nitrogen fertilizer alone, the top three factors influencing crop yield (Figure 15c) and nitrogen fertilizer productivity (Figure 15d) are nitrogen application rate (26.69%, 35.27%), crop type (20.45%, 27.38%), and annual precipitation (15.96%, 20.84%).

## 3. Discussion

### 3.1. Effects of MNF on Crop Yield and PFPN

In the new era of agricultural production under the background of “reducing nitrogen fertilizer usage and increasing efficiency, innovating development”, fully tapping into the potential of nitrogen fertilizer for increasing yield and improving quality, reducing nitrogen fertilizer usage and environmental pollution, is a major challenge facing the world [13,43]. MNF can fully leverage the advantages of urea and slow-release nitrogen fertilizers, promote sustained and balanced nitrogen supply, and steadily improve crop yield and PFPN [17,18]. This study found that compared with Urea, the crop yield and PFPN of MNF significantly increased by 7.42% and 8.20%, respectively (Table 1). This is consistent with the research results of Zhang et al. [44] and Zou et al. [45]. Compared with the C/SRF, the crop yield significantly increased by 2.44% with the MNF, but the effect of PFPN improvement was not significant (Table 1). It can be seen that the MNF has a better effect on improving PFPN and yield than the urea, while the C/SRF has a lower effect. The main reason for this is: (1) urea is a high-concentration and quick-acting nitrogen fertilizer. After application, nitrogen is completely released in a short period of time, which cannot provide a sufficient nitrogen supply for the entire growth period of crops. In addition, the rapid increase in soil nitrogen concentration will exacerbate soil acidification and compaction, affect root development, and lead to low crop production levels [22,37]. The nitrogen release characteristics after MNF can meet the nitrogen absorption requirements of crops, ensuring nitrogen supply during the critical period of crop growth and preventing fertilizer loss in the later stage of crop growth. Therefore, compared with urea, it can effectively improve crop productivity [23]. (2) C/SRF can control the slow release of nitrogen by changing the diffusion flux between the core urea particles and the boundary environment, prolong the nitrogen release time, and ensure the nitrogen supply in the later stage of crop growth. It also shows a certain increase in yield and PFPN in agricultural production [46,47]. However, for crops with high nitrogen demand in the early growth stage and long growth period, C/SRF may result in insufficient nitrogen supply. Therefore, the MNF can improve crop production level compared to C/SRF. However, the current technology of MNF cannot fully match the nitrogen release law with the nitrogen demand law of crops. Therefore, the productivity improvement effect of MNF is lower than that of C/SRF, and there is still great potential for exploration [14,15].

### 3.2. Factors Affecting the Effects of MNF on Crop Yield and PFPN

#### 3.2.1. Management Factor

Scientific and reasonable management measures can significantly improve crop growth and production conditions, while protecting soil health and the environment [18]. The nitrogen content, release period, type, blending ratio, and N rate of slow-release nitrogen fertilizer significantly affect the effect of MNF. This study found that compared with the urea and C/SRF, the use of control-release nitrogen fertilizer with an N content of 25–30% mixed with urea resulted in the best increase rate in crop yield and PFPN (Figure 3a,b and Figure 9a,b). This may be because the nutrient release curve of the slow-release nitrogen fertilizer mixed with this nitrogen content is consistent with the nitrogen demand pattern of most crops, which can achieve efficient nutrient utilization. This study found that compared with the urea and C/SRF, the crop yield and PFPN increase rate of MNF were higher when the release cycle was 80–100 days and 40–80 days, respectively (Figure 3a, Figure 9a, Figure 3b, Figure 9b). Mainly because the release cycle is different, the nitrogen release pattern is not the same, which affects the nitrogen absorption and utilization efficiency of crops [48,49].

The blending ratio of slow- and controlled-release nitrogen fertilizer is not the higher the better; the reasonable blending ratio can make the MNF maximize the quality and yield effect and economic benefits. This study found that as the blending ratio increased, the crop yield and the PFPN increase rate of MNF showed a trend of first increasing and then decreasing. Compared with the urea and C/SRF, the crop yield and PFPN improvement effect of MNF are better when the blending ratio is 0.3–0.7 (Figure 4a,b and Figure 10a,b). This is consistent with the conclusions of Fan et al. [49] and Zhang et al. [50] in their studies on rice, wheat, and corn. Perhaps because too high or too low a mixing ratio can weaken the advantages of MNF, its crop production effect is close to that of urea or C/SRF [47]. This study found that mixed application of CRF had a better effect on improving crop yield and PFPN than SRF (Figure 4a,b and Figure 10a,b). This is because SRF utilizes chemical and biological technologies to slow down the rate of nutrient release. For example, sulfur-coated urea involves coating the surface of urea granules with a layer of sulfur and sealing it with wax. Water penetrates through the micro-pores in the coating to dissolve the nutrients inside, and the release rate is influenced by factors such as soil pH, microbial activity, and moisture conditions [51]. On the other hand, CRF uses a water-soluble outer coating to slowly release nutrients from within the membrane. The release rate is primarily influenced by soil temperature, making the nitrogen release from CRF more stable and efficient [44].

In order to pursue high yield, excessive fertilization is common in agricultural production, which can lead to a decrease in crop yield, nitrogen fertilizer utilization efficiency, and soil fertility. This study found that the N rate is the main factor affecting the effects of MNF on crop yield and PFPN. The crop yield and PFPN increase rate of MNF showed a trend of first increasing and then decreasing with the increase in N rate. In the combination of MNF vs. urea, the crop yield and PFPN increase rate were higher when the N rate was 150–240 kg·ha^−1^ (Figure 4b and Figure 10b). In the combination of MNF and C/SRF, the crop yield and PFPN increase rate were higher when the N rate was 150–270 kg·ha^−1^ (Figure 4d and Figure 10d). Similarly, Xue et al. [41] reached consistent conclusions in their studies on potatoes, and Jiang et al. [52] on rice. This is because low N rates can limit crop photosynthetic efficiency, leaf area index, and dry matter accumulation, hindered the formation of yield, high N rates can lead to excessive growth of crop nutrition stages, increase the risk of pests and diseases, hinder nutrient transfer to grains, and cause soil compaction, soil acidification, and soil-borne diseases, affecting crop growth [22,24,28].

#### 3.2.2. Regional Factor

China has a vast territory, ranging from cold regions in the north to tropical regions in the south, from coastal plains in the east to plateaus and mountains in the west, creating a unique natural geographical environment and complex and diverse climate types. The crop production effect of MNF has obvious regional characteristics. This study found that in the combination of MNF vs. urea, the crop yield and PFPN of MNF increased at higher rates and were not significantly different in Northwest, North, and Northeast China (Figure 5a and Figure 11a), which is consistent with the research results of Zheng et al. [53]. The northwest region is facing problems such as secondary soil salinization, water scarcity, and severe soil erosion, which have accelerated the degradation of soil fertility [44]. The northeast region has shallow cultivation layers, high bulk density, loose and compact soil structure, and a high multiple cropping index [54]. The North China region is mainly dominated by irrigated agriculture, with severe soil compaction [55]. The MNF has effectively improved the soil nutrient and structural deficiencies, as well as the current status of water and fertilizer management in these three regions. In the combination of MNF and C/SRF, the crop yield and PFPN increase rate of MNF were significantly reduced in South China (Figure 5c and Figure 11c). This is similar to the research findings of Zhu et al. [56]. This is because South China belongs to a tropical monsoon climate, where high temperatures and high humidity accelerate the decomposition rate of nitrogen fertilizers and weaken the fertilizer efficiency of MNF [57]. In addition, contrary to the research findings of Meng et al. [55], this study found that the MNF in the southwest region had no significant effect (Figure 5c and Figure 11c). This may be due to the fact that this study only involved some southwest provinces (Yunnan, Guizhou, and Sichuan) and did not fully reflect the application effects of MNF in the southwest region.

Temperature and precipitation are important climatic factors that affect agricultural production. Excessive temperature can lead to strong transpiration of crops, making them susceptible to drought; low temperatures can slow down crop growth, reduce respiration, and cause photosynthetic stress and intensity [58,59]. Excessive precipitation can accelerate nitrogen transport, increase leaching and runoff losses, affect root development, and reduce nutrient absorption and utilization efficiency [60]. Low precipitation can affect the dissolution rate of C/SRF, leading to an imbalance in nitrogen supply [61]. This study found that compared with urea, the crop yield and PFPN increase rate of MNF application were higher when the AAT was ≤20 °C (Figure 5a and Figure 11a); when the AAP is 400–800 mm, the crop yield increase rate is higher, and when the AAP is 200–400 mm, the improvement rate of crop PFPN is higher (Figure 5b and Figure 11b). Mainly because the suitable growth temperature for most crops is 15~25 °C, under which soil microbial activity is high, which is conducive to the dissolution of C/SRF and enzymatic conversion of soil nutrients [62]. When the AAP is 400–800 mm, it can provide sufficient water for crops and promote crop yield formation. However, high precipitation can easily cause deep nitrogen leakage, which is not conducive to improving nitrogen use efficiency [63]. Compared with C/SRF, when the AAT is ≤5 °C, the crop yield and PFPN increase rate of MNF are higher (Figure 5d and Figure 11d). This may be due to the slow nutrient release rate of C/SRF when the temperature is low, and the difficulty of nutrient dissolution and diffusion in the soil. The MNF can precisely supplement the nitrogen demand of crops in the early stage of growth.

Altitude indirectly affects crop growth and development by changing conditions such as temperature, humidity, and light. This study found that the crop yield and nitrogen partial productivity increase rate of MNF showed a trend of first increasing and then decreasing with altitude. Compared with urea, the crop yield and PFPN increase rate of MNF were higher at altitudes of 750–1050 m (Figure 5b and Figure 11b). However, the number of samples in this range is relatively small, and further research is needed to determine the appropriate altitude range. Compared with the C/SRF, the crop yield and PFPN increase rate of MNF were higher at altitudes of 300–900 m (Figure 5d and Figure 11d). Bhandari et al. [62] also reached similar conclusions in their research on sand seed cultivation. This is because the temperature gradually decreases with increasing altitude, which affects the growth rate and cycle of crops. In areas above 1000 m above sea level, precipitation generally increases, and high-humidity environments can limit the nitrogen release rate of C/SRF, which is not conducive to crop growth [63,64].

#### 3.2.3. Soil Factor

The soil texture determines the water storage, fertilizer retention, and air permeability of the soil. Thereby affecting crop growth [19]. This study found that there was no significant difference in crop yield and PFPN increase rate between MNF and urea in soils of different textures (Figure 6a and Figure 12a). This indicates that, in different types of soils, MNF can stably increase crop yield and PFPN compared to urea and has broad application prospects. In the combination of MNF vs. C/SRF, the increase rate in crop yield and PFPN of MNF was better in sandy soil, not significant in loam soil, and significantly increased in yield, but PFPN was not significant in clay soil (Figure 6b and Figure 12b). This may be due to the large particle size and high porosity of sandy soil, which has poor water and fertilizer retention performance. The nitrogen supply of MNF can better match the nitrogen demand of crops, reducing nitrogen loss [65]. The particles and porosity of loam soil are moderate, and it has strong water and fertilizer retention ability. The advantage of C/SRF is not obvious when MNF [66]. Clay particles are small, with low porosity and slow nitrogen release and infiltration rates. MNF can increase nitrogen supply, thereby improving crop yield. However, clay adsorption is strong and may affect nitrogen fixation and transformation [55].

This study found that compared with the urea and C/SRF, the crop yield and PFPN improvement of PFPN were higher at soil pH 5.5–6.5 and soil pH > 7.5, respectively (Figure 6a, Figure 12a, Figure 6b, Figure 12b). Feng et al. [65] also reached a consistent conclusion. Perhaps because when the soil pH is 5.5–6.5, the abundance and activity of soil micro-organisms are higher, which can promote the decomposition of soil organic matter and nutrient transformation [67,68,69]. When the soil pH is ≥7.5, the solubility and availability of some nutrients will decrease, and the nitrification of nitrogen fertilizer will also be inhibited. MNF can provide different types of nitrogen sources, reduce the absorption of nitrogen by microbial denitrification populations, improve nitrification efficiency, and ensure that more nitrogen is absorbed and utilized by crops [68,69,70].

#### 3.2.4. Crop Type

The nitrogen demand characteristics of crops are synergistically regulated by genotype, growth period, and growth habit [44]. This study found that compared with urea, the yield and PFPN increase rate of MNF were higher in rapeseed, corn, and cotton, and lower in soybean and rice (Figure 7a and Figure 13a). This may be due to the similarity in nitrogen requirements among rapeseed, corn, and cotton. Rapeseed has a longer growth period, developed roots, and strong nitrogen absorption capacity; cotton is a crop with infinite inflorescences, and its nutritional and reproductive growth periods overlap for a long time, requiring a large amount of nitrogen throughout the entire growth period; corn is a C4 crop, and its high photosynthetic carbon assimilation efficiency drives a strong demand for nitrogen metabolism [23,71]. Compared with Urea, the nitrogen release pattern of MNF is highly consistent with the nitrogen requirements of rapeseed, corn, and cotton, thereby significantly improving their yield and PFPN. Soybeans are leguminous plants that can utilize rhizobia for nitrogen fixation and have a lower demand for exogenous nitrogen [72]. Rice is a typical aquatic crop, and long-term high water content environments can affect nitrogen release rates, thereby weakening the effectiveness of MNF [37]. Compared with the C/SRF, the MNF has a better effect on improving yield and MNF in potatoes and rapeseed; the improvement effect is relatively low in corn and cotton; the increase in wheat is not significant. This may be related to factors such as the type of C/SRF, mixing ratio, and N rate.

#### 3.2.5. Time Factor

The United States was the first country to develop C/SRF. China started developing C/SRF relatively late and only formed a relatively complete C/SRF production chain in 2006. From the distribution of the data samples in this study, it can also be seen that there were only eight samples before 2005. This study found that in the combination of MFN vs. urea, the crop yield increase rate of MNF showed an upward trend before 2015. Starting from 2016, the crop yield increase rate of MNF gradually decreased, while the increase rate of PFPN of crops under MNF was exactly the opposite (Figure 8a and Figure 14a). This may be related to national policies and nitrogen fertilizer application status. In 2004, China formulated the policy of “industry supporting agriculture and cities supporting rural areas”, and since then, crop yields have increased for twelve consecutive years. Since 2016, the country has initiated a supply-side structural reform in agriculture, resulting in a reduction in the planting area of grain crops. In 2015, the amount of nitrogen fertilizer used in China reached 29.68 million tons, a historic high. The growth rate of grain yield slowed down, and the nitrogen fertilizer utilization efficiency significantly decreased [73]. During the 13th Five Year Plan period, China introduced the “Action Plan for Zero Growth in Fertilizer Use by 2020” and the “Guiding Opinions on Promoting the Transformation and Development of the Fertilizer Industry”, effectively reducing the amount of nitrogen fertilizer used in China. In 2016, China’s nitrogen fertilizer usage experienced negative growth for the first time, effectively improving nitrogen fertilizer utilization efficiency. In the combination of MNF and C/SRF, before 2011, the crop yield increase rate of MNF showed a negative effect and then showed an upward trend; before 2016, the MNF had a negative effect on crop nitrogen partial productivity, and the improvement rate showed an upward trend thereafter (Figure 8b and Figure 14b). This may be because the MNF technology is not yet mature in the early stages, and the application effect has not been fully utilized. As research deepens and experience accumulates, the advantages of MNF gradually become prominent [40,41].

The application of nitrogen fertilizer is the most common agronomic measure in agricultural ecosystems, and its crop production effect is closely related to the application period [74]. This study found that compared with urea, the crop with MNF had a better effect on improving yield and PFPN after 1–2 years of planting (Figure 8a and Figure 14a). Perhaps because planting the same crop continuously on the same farmland can disrupt the dynamic balance of the soil-crop system and create continuous cropping obstacles [75]. Compared with the C/SRF, the crop with MNF showed a higher increase in yield and PFPN after 3 years of planting (Figure 8b and Figure 14b). This may be related to the long fertilizer efficiency of C/SRF [53].

### 3.3. Limitations and Future Directions

When conducting a meta-analysis, selecting high-quality studies can enhance the reliability of the research; however, it is challenging to include all relevant studies, thereby limiting the scope and completeness of the database used in this study. Additionally, the effects of nitrogen fertilizer application are influenced by the interaction of multiple factors. When investigating the impact of individual factors on the effectiveness of nitrogen fertilizer application, it is difficult to exclude the influence of other factors, such as fertilization practices, experimental personnel, and experimental background parameters. Furthermore, this study only examined the effects of nitrogen fertilizer mixing on crop yield and nitrogen fertilizer productivity. However, nitrogen fertilizer mixing also impacts greenhouse gas emissions from farmland, and the membrane material of controlled-release fertilizers can affect the soil environment. Future research should strengthen investigations into these aspects.

## 4. Materials and Methods

### 4.1. Data Sources and Screening

By conducting searches in both Chinese and English databases, including the China National Knowledge Infrastructure (CNKI), Scopus, Wiley, and Google Scholar, and Web of Science, research papers on the effects of MNF on crop yield and PFPN published before July 1, 2024, were collected. The Chinese search terms used were “slow-release fertilizer”, “controlled-release fertilizer”, “urea” and “yield”; the corresponding English search terms included “slow release fertilizer”, “controlled release fertilizer”, “urea” and “yield”. To improve the precision of the research results, samples were screened based on the following criteria: (1) The study site is located in China, and the experiments were conducted under open-field conditions; (2) the experimental treatments must simultaneously include both MNF and urea, or MNF and C/SRF, or MNF, urea and C/SRF, with the same nitrogen application rates (calculated as pure nitrogen input) and consistent management practices; (3) information on the experimental location, nitrogen management, and crop type is clearly documented; (4) data on crop yield and PFPN can be directly or indirectly obtained. After applying these criteria, a total of 772 samples were collected for MNF vs. urea, 400 samples were collected for MNF vs. C/SRF, respectively (Table 2). During the data collection process, if the required data were presented in graphical form, the software Get Data Graph Digitizer 2.2.6 was used for extraction. If the same experimental data appeared in multiple publications, it was included in the analysis only once.

### 4.2. Data Classification

**Figure 16:**
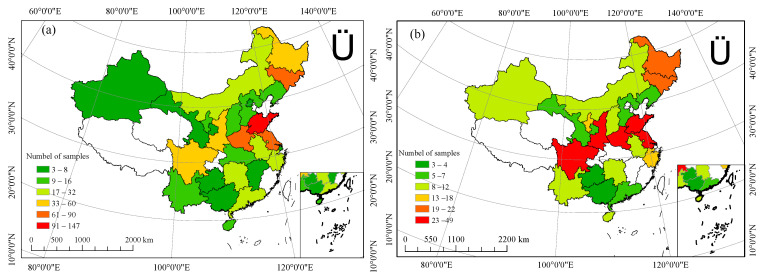
Sample distribution. (**a**) shows the sample distribution of MNF compared to urea, (**b**) shows the sample distribution of MNF compared to C/SRF.

The samples included in this study cover 26 provinces (Figure 16), autonomous regions, and municipalities, representing most crop-growing areas in China. The data samples are highly representative. Given the wide geographic distribution of the samples and considering administrative divisions, climatic characteristics, and similar farming systems, the study area was divided into seven regions: East China (Shanghai, Shandong, Jiangxi, Zhejiang, Anhui, Jiangsu), North China (Beijing, Hebei, Shanxi, Inner Mongolia Autonomous Region), Central China (Hubei, Hunan, Henan), Northwest China (Shaanxi, Gansu, Ningxia Hui Autonomous Region, Xinjiang Uygur Autonomous Region), Southwest China (Guizhou, Yunnan, Sichuan), South China (Guangxi Zhuang Autonomous Region, Hainan, Guangdong), and Northeast China (Heilongjiang, Jilin, Liaoning).

To better reflect the effects of nitrogen fertilizer on crop yield and productivity, as well as the main influencing factors, this study refers to previous research. This study categorizes the key indicators influencing the effects of nitrogen fertilizer application into six categories (Table 3): fertilizer characteristics (nitrogen content and nitrogen release period), fertilizer management (blending ratio, nitrogen fertilizer type, and nitrogen application rate), regional factor (geographical location, annual average precipitation, annual average temperature, and altitude), soil factors (soil texture and soil pH), crop factor, and time factor (fertilization period, experiment year, and growth year). Grouping was carried out by considering the number and distribution of samples for each influencing factor. The characteristics of fertilizers are classified according to the Chinese national standard GB/T 23348-2009 for Slow-release Fertilizers and the chemical industry standard HG/T 4215-2011 for Controlled-release Fertilizers [76,77]. The indicators for nitrogen fertilizer management are grouped based on the literature [78,79] and the distribution characteristics of the sample data in this study. Annual precipitation is divided into four groups: arid, semi-arid, semi-humid, and humid regions; annual temperature is categorized in 5 °C intervals; elevation is categorized in 150 m intervals; soil texture and soil pH are referenced according to the U.S. Department of Agriculture (USDA) soil texture classification standard and the Technical Guidelines for the Classification of Farmland Soil Environmental Quality issued by the Chinese Ministry of Agriculture. The experimental years align with the start and end dates of the national five-year plan; since fertilization timing only involves basal application and top dressing, no grouping is performed.

### 4.3. Data Analysis

#### 4.3.1. Calculation of PFPN

The nitrogen partial factor productivity (PFPN) of crops was calculated using Equation (1):

(1) $$PFPN=\frac{Y}{N}$$

where *Y* (Yield, kg·ha^−1^) is the crop yield of the treatment or control group, and *N* (kg·ha^−1^) is the N rate (quantity of pure nitrogen) in the treatment or control group.

#### 4.3.2. Standard Deviation Calculation

Standard deviation (*SD*) is an important parameter in meta-analysis, used to calculate the weight of each sample. When the literature lists the standard deviation of yield (PFPN) for the treatment in question, it is used directly; if the data provided in the literature are standard errors (*SE*), they are converted to standard deviations (*SD*) through Equation (2):

(2) $$SD=\frac{SE}{\sqrt{n}}$$

where *n* is the number of experimental replicates.

#### 4.3.3. Effect Size Calculation

Effect size is a metric used to measure the magnitude of treatment effects. It was calculated using the response ratio as shown in Equation (3):

(3) $$lnR=ln(\frac{X_{t}}{X_{c}})$$

where *R* is the response ratio, *X_t_* is the average crop yield and PFPN in the treatment group, and *X_c_* is the average crop yield and PFPN in the control group.

To more intuitively reflect the effects of MNF on crop yield and PFPN, the effect size *lnR* was converted to an increase (or improvement) rate Z using Equation (4):

(4) $$Z(100\%)=\left[exp(lnR-1)\right]\times 100$$

The weighted effect size (*lnR*′) and confidence interval (*CI*) were calculated using Equations (5)–(8):

(5) $$\vartheta =\frac{{SD}_{t}^{2}}{\left(n_{t}X_{t}^{2}\right)}+\frac{{SD}_{c}^{2}}{n_{c}X_{c}^{2}}$$

(6) $$\omega_{i}=\frac{1}{\vartheta }$$

(7) $$lnR^{\prime }=\frac{lnR_{i}\omega_{i}}{K}$$

(8) $$95\%CI=lnR^{\prime }\pm 1.96S$$

In the formula, ϑ is the variance (statistics), ${SD}_{t}^{2}$ and ${SD}_{c}^{2}$ are the standard deviations of crop yield (PFPN) in the treatment and control groups, respectively, and nt and nc are the sample numbers of crop yield (PFPN) in the treatment and control groups, respectively. $\omega_{i}$ and $lnR_{i}$ are the weight and effect size of the i-th sample, respectively. *K* is the number of samples and *S* is the standard deviation.

If the 95% *CI* of *Z* is entirely greater than 0, it indicates that MNF has a significant positive effect on crop yield (PFPN). If it is entirely less than 0, it indicates a significant negative effect. If the confidence interval includes 0, it indicates that MNF has no significant effect on crop yield (PFPN).

#### 4.3.4. Heterogeneity Test

To determine whether there is heterogeneity among the samples (i.e., whether the variations in results among different samples are due to random errors), the *Q*-statistic was used to calculate heterogeneity using Equation (9):

(9) $$Q=\frac{\sum_{i=1}^{K}\omega_{i}{\left(lnR_{i}\right)}^{2}-{\left(\sum_{i=1}^{K}\omega_{i}{lnR}_{I}\right)}^{2}}{\sum_{i=1}^{K}\omega_{i}}$$

If the test result is *p* > 0.05 (significance test *p*-value of the *Q*-statistic), it indicates homogeneity among different treatments or study results, and a fixed-effect model is used to calculate the combined statistics. Otherwise, a random-effect model is adopted.

#### 4.3.5. Publication Bias Test

Publication bias is a common small-sample effect that reduces the accuracy of analysis results. In this study, publication bias was assessed using an inverted funnel plot and Egger’s test. If *p* > 0.05, no publication bias was considered to exist; if *p* < 0.05, Rosenthal’s tolerance number was calculated. If the tolerance number > 5 × n + 10, it indicates that there was no publication bias in the study results [80].

#### 4.3.6. Random Forest Model

Random Forest model is a powerful machine learning algorithm based on ensemble learning. By constructing and combining multiple decision trees, it can handle complex data relationships, robustness, overfitting resistance, feature importance, and ease of use. Its basic framework includes autonomous sampling, random feature selection, parallel construction of decision trees, and ensemble prediction [81]. Given the objective of this study to identify the key factors influencing the effects of nitrogen fertilizer application, combined with the data characteristics of this study, the random forest model demonstrates strong applicability.

### 4.4. Data Processing

Statistical analysis was performed using R software (v.4.1.0). Meta-analysis was conducted using the “metaphor” package. To assess the relative importance of each influencing factor, a random forest model was constructed using the “randomForest” package for analysis. All charts were created using Origin 2021 software. Statistical analysis was set with α = 0.05 as the threshold for statistical significance.

## 5. Conclusions

The crop yield and PFPN of MNF were significantly increased by 7.42% and 8.20%, respectively, compared to urea, and significantly increased by 2.44% and not significantly increased, respectively, compared to C/SRF.

In the combination of MNF and urea, compared to the application of urea alone, in Northwestern China, where the annual average temperature is ≤20 °C, annual precipitation is 200–800 mm, altitude is 750–1050 m, and soil pH is 5.5–6.5, the use of controlled-release nitrogen fertilizers with a nitrogen content of 25–35%, with a mixing ratio of ≥0.3, with nitrogen application rates of 150–240 kg·ha^−1^, the best yield and nitrogen fertilizer productivity enhancement effects are achieved when nitrogen fertilizer is applied in a mixed ratio for 1–2 years when growing rapeseed, corn, and cotton. The main factors affecting the effect of mixed nitrogen fertilizer application are crop type, nitrogen application rate, and soil pH.

Compared to the application of C/SRF alone, in Northwestern China, where the annual average temperature is ≤5 °C, annual average precipitation is ≤400 mm, elevation is 300–900 m, and soil pH is >7.5, the use of controlled-release nitrogen fertilizers with a nitrogen content of 25–30% and a release period of 40–80 days, applied at a mixing ratio of 0.4–0.7, applying 150–270 kg·ha^−1^ of nitrogen, and growing potatoes or rapeseed for three years, both high yields and improved PFP rates can be achieved simultaneously. The primary factors influencing the mixed application effect of nitrogen fertilizers are nitrogen application rate, crop type, and annual precipitation.

Overall, the application of controlled-release nitrogen fertilizer alone is superior to the application of urea alone in terms of improving crop yield and PFPN. Furthermore, when controlled-release nitrogen fertilizer and urea are mixed in a ratio of 0.4–0.7, the synergistic effect on yield and PFPN is even better.

## Figures and Tables

**Table 1:** Overall effect of MNF on crop yield and PFPN.

Index	Treatment	Model	Increase Rate (%)	95% CI (%)		Effect Size Test		Heterogeneity Test		Publication Test	
				*LL*	*UL*	*Z*	*P*	*Q*	*P_Q_*	*Z_B_*	*P_B_*
Yield	MNF vs. Urea	REM	7.42	6.49	8.36	16.12	<0.0001	26,544.49	<0.0001	0.54	0.59
	MNF vs. C/SRF	REM	2.44	1.24	3.66	4.012	<0.0001	25,579.75	<0.0001	0.84	0.40
PFPN	MNF vs. Urea	REM	8.20	7.22	9.87	13.17	<0.0001	337,679.9	<0.0001	0.36	0.91
	MNF vs. C/SRF	REM	0.24	−0.63	3.20	0.16	0.8705	34,082.89	<0.0001	0.78	0.44

*LL*: Lower limit; *UL*: Upper limit; REM: Random effect model; *Z*: The statistic of effect size; *P*: The significant value of the effect size; *Q*: The statistic of heterogeneity; *P_Q_*: The significant value of *Q*; *Z_B_*: The statistic of publication bias; *P_B_*: The significant value of the publication bias (*Z_B_*).

**Table 2:** Data sample.

Combination	Treatment Group	Control Group	Number of Studies	Number of Samples
MNF vs. Urea	MNF	Urea	137 (125 in Chinese, 12 in English)	772
MNF vs. C/SRF	MNF	C/SRF	94 (90 in Chinese, 4 in English)	400

MNF: Mixed nitrogen fertilizer; C/SRF: Slow and controlled release nitrogen fertilizer.

**Table 3:** Indicator classification.

Influence Factor	Index	Group									
		1	2	3	4	5	6	7	8	9	10
Characteristics of nitrogen fertilizer	N content (%)	≤25	25–30	30–35	35–40	40–45	≥45	—	—	—	—
	RP (d)	≤40	40–60	60–80	80–100	100–120	>120	—	—	—	—
Nitrogen fertilizer management	NFT	CRF	SRF	—	—	—	—	—	—	—	—
	N rate (kg·hm^−2^)	≤90	90–120	120–150	150–180	180–210	210–240	240–270	270–300	>300	—
	BR	≤0.1	0.1–0.2	0.2–0.3	0.3–0.4	0.4–0.5	0.5–0.6	0.6–0.7	0.7–0.8	≥0.8	—
Region factor	Area	East China	North China	Northwest	Southwest	South China	Central China	Northeast	—	—	—
	AAP (mm)	≤200	200–400	400–800	≥800	—	—	—	—	—	—
	AAT (°C)	≤5	5–10	10–15	15–20	20–25	≥25	—	—	—	—
	Elevation (m)	≤150	150–300	300–450	450–600	600–750	750–900	900–1050	1050–1200	≥1200	—
Soil factor	ST	Loam	Sandy	Clay	—	—	—	—	—	—	—
	Soil pH	<6.5	6.5–7.5	>7.5	—	—	—	—	—	—	—
Crop type	Crop type	Maize	Rice	Wheat	Cotton	Rapeseed	Tomato	Potato	Scallion	Soybean	Others
Time factor	Fertilization period	Base fertilizer + top dressing		—	—	—	—	—	—	—	—
	EY	≤2005	2006–2010	2011–2015	2016–2019	≥2020	—	—	—	—	—
	GY	1	2	3	4	—	—	—	—	—	—

EY: Experiment year; GY: Growth year; AAP: Annual average precipitation; AAT: Annual average temperature; ST: Soil texture; NFT: Nitrogen fertilizer type; CRF: Controlled release fertilizer; SRF: Slow-release fertilizer; RP: Release period; BR: Blending ratio; CT: Crop type. Since the fertilization period only includes basal fertilization and top dressing, and the conclusions regarding fertilization timing are consistent, no meta-analysis is performed.

## Data Availability

All data supporting this study are included in the article.
